# GelMA synthesis and sources comparison for 3D multimaterial bioprinting

**DOI:** 10.3389/fbioe.2024.1383010

**Published:** 2024-03-25

**Authors:** Cesare Gabriele Gaglio, Désireé Baruffaldi, Candido Fabrizio Pirri, Lucia Napione, Francesca Frascella

**Affiliations:** ^1^ Department of Applied Science and Technology (DISAT)—PolitoBIOMed Lab—Politecnico di Torino, Turin, Italy; ^2^ Center for Sustainable Future Technologies, Italian Institute of Technology, Turin, Italy

**Keywords:** multimaterial 3D printing, bioprinting, GelMA bioink, scaffold, sacrifical polymer

## Abstract

Gelatin Methacryloyl (GelMA) is one of the most used biomaterials for a wide range of applications, such as drug delivery, disease modeling and tissue regeneration. GelMA is obtained from gelatin, which can be derived from different sources (e.g., bovine skin, and porcine skin), through substitution of reactive amine and hydroxyl groups with methacrylic anhydride (MAA). The degree of functionalization (DoF) can be tuned by varying the MAA amount used; thus, different protocols, with different reaction efficiency, have been developed, using various alkaline buffers (e.g., phosphate-buffered saline, DPBS, or carbonate-bicarbonate solution). Obviously, DoF modulation has an impact on the final GelMA properties, so a deep investigation on the features of the obtained hydrogel must be carried on. The purpose of this study is to investigate how different gelatin sources and synthesis methods affect GelMA properties, as literature lacks direct and systematic comparisons between these parameters, especially between synthesis methods. The final aim is to facilitate the choice of the source or synthesis method according to the needs of the desired application. Hence, chemical and physical properties of GelMA formulations were assessed, determining the DoFs, mechanical and viscoelastic properties by rheological analysis, water absorption by swelling capacity and enzymatic degradation rates. Biological tests with lung adenocarcinoma cells (A549) were performed. Moreover, since 3D bioprinting is a rapidly evolving technology thanks to the possibility of precise deposition of cell-laden biomaterials (bioinks) to mimic the 3D structures of several tissues, the potential of different GelMA formulations as bioinks have been tested with a multi-material approach, revealing its printability and versatility in various applications.

## 1 Introduction

3D cell culture-based research experienced an exponential growth in the last 30 years (Jensen and Teng, 2020; Ding et al., 2023). One of the most advanced techniques to produce 3D cell cultures is 3D bioprinting, a rapidly rising technology, that allows researchers to recreate *in vitro* models and engineered tissues for a plethora of applications, ranging from regenerative medicine, 3D organ bioprinting for transplantation, high-throughput screening of drug and toxicology screening (Bejoy et al., 2021). Moreover, both healthy and diseased models can be reproduced (Walus et al., 2020). Among the different technologies introduced (Papaioannou et al., 2019), extrusion-based bioprinting (EBB) takes a special place in consideration as one of the most used (Ramesh et al., 2021) and consists in the deposition of bioink, a formulation composed of an acellular biomaterial embedded with cells (Groll et al., 2019). EBB offers the possibility to perform multi-material printing with multiple independent printheads (Ravanbakhsh et al., 2021). A wide range of materials can be printed at once, such as hydrogels, sacrificial inks or thermoplastic materials (B. S. Kim et al., 2019; Kolesky et al., 2016), leading to a further level of complexity of the printed models. For example, this technique can address the common issue of the absence of vasculature in most of the 3D models developed so far (E. P. Chen et al., 2021; Ileiwat et al., 2022). Hydrogels have proved to be the optimal choice as bioink materials (Xie et al., 2023), due to their biocompatibility, biodegradability and remarkable capacity to trap huge amounts of water, improving the transportation of O_2_ and nutrients (Saroia et al., 2018). Furthermore, they can support cell adhesion and proliferation when they have a structure similar to the extracellular matrix (ECM) in which cells are embedded *in vivo* (González-Díaz and Varghese, 2016).

Hydrogels can be classified as synthetic, natural, or they can be derived from a natural source and further chemically modified to provide more stable mechanical properties, of which natural polymers generally lack (X. B. Chen et al., 2023). Chemical modifications include grafting of chemical groups such as vinyl sulfone or methacrylic acid to promote hydrogel formation or with peptide sequences such as RGD to enhance cellular adhesion (Baruffaldi et al., 2021). Methacrylation, in particular, allows to introduce photosensible groups to the side chains of different natural polymers, such as silk (S. H. Kim et al., 2018), hyaluronic acid (Spearman et al., 2020) or gelatin (Shi et al., 2023), improving their mechanical properties.

Gelatin Methacryloyl (GelMA), one of the most widely used bioink (Shi et al., 2023), is obtained by reacting gelatin and methacrylic anhydride (MAA). Different sources of gelatin are commonly used, however, the most employed in bioengineering applications are Type B bovine gelatin (Pahoff et al., 2019; Vassallo et al., 2022; Villata et al., 2023) and Type A porcine gelatin (González-Gamboa et al., 2022; J. Y. Lee et al., 2023; Nagaraj et al., 2022; Paul et al., 2023). In 2000, Van Den Bulcke et al. (Van Den Bulcke et al., 2000) proposed the first synthesis method for GelMA, which involved the use of PBS as solvent and a simple dropwise addition of MAA to the heated solution. Subsequently, many researchers tried to improve the reproducibility of the synthesis and to optimize the amount of used MAA. First, a sequential addition of the MAA at a precise rate was proposed (Nichol et al., 2010), then the buffer was changed to a sodium carbonate-bicarbonate (CB) with a pH of 9.6, that was adjusted after every MAA addition to enhance the reaction efficiency (B. H. Lee et al., 2015).

Finally, Shirahama at Al (Shirahama et al., 2016) presented a One-pot Method, using CB buffer (pH 9.4), and adjusting the pH before a single dropwise addition of MAA to the heated gelatin solution. The latter method sensibly reduces the amount of MAA used and guarantee a better batch to batch consistency of the synthesized GelMA (Zhu et al., 2019). However, to the best of our knowledge, there is currently no direct comparison between GelMA obtained with different protocols.

In this study, various aspects of the synthetized GelMA will be analyzed by chemical, mechanical and biological characterizations, starting with addressing the effects of a pre-filtration step, used in some case studies (Shirahama et al., 2016; Ko et al., 2019; Zhu et al., 2019; Zhou et al., 2022). Then, the effects of the One-Pot method on GelMA synthesis using different gelatin sources (Type A from porcine skin, Type B from bovine skin) and different Degrees of Functionalization (DoF) were characterized with a focus on the rheological properties of the material. Finally, a comparison between the first method introduced by Van Den Bulcke and the One Pot method developed by Shirahama and further refined by Zhu will be presented, to compare the first method introduced and the one that offers the most refinement in terms of MAA quantity optimization, reduction of the numbers of operation and reproducibility.

The last section is dedicated to the feasibility to combine the printing of different GelMA formulations and Pluronic F-127, a thermosensitive polymer known for its optimal performance as sacrificial ink (S. Liu et al., 2022). One crucial aspect, to reach good level of resolution in multimaterial 3D bioprinting, is the compatibility between the materials that are printed in a single printing session. Therefore, in this study, an investigation was carried to print GelMA scaffold with hollow, patent channels inside with a relatively small size (<400 µm) that could be further used to replicate a perfusable vascular network in following studies.

## 2 Materials and methods

### 2.1 GelMA synthesis

Gelatin Methacryloyl (GelMA) was synthesized by following two previously reported protocols (B. H. Lee et al., 2015; Van Den Bulcke et al., 2000) and it was derived from type B gelatin from bovine skin (Bloom 50-120, Sigma Aldrich, G6650) and type A gelatin from porcine skin (Bloom 300, Sigma Aldrich, G2500).

The first method used, referred to as “Method 1,” was firstly described by Van Den Bulcke (Van Den Bulcke et al., 2000) and, briefly, consisted in the dissolution of 10 g of gelatin into 100 mL of Dulbecco’s Phosphate Buffered Saline (DPBS, Sigma Aldrich, D1408) to obtain a concentration of 10% w/v at 50°C for 1 h. To introduce methacryloyl groups to gelatin’s reactive amine and hydroxyl groups, different amounts of Methacrylic Anhydride (MAA, Sigma Aldrich, 276685) were added dropwise under continuous stirring, specifically 2 mL, 4 mL and 8 mL to obtain low, medium or high degree of functionalization (DoF). The reaction lasted 2 h in the dark at 40°C under magnetic stirring, then was stopped by diluting the reaction mixture with an equal volume of DPBS (i.e., 100 mL). The resulting solution was, then, dialyzed against ddH_2_O with cellulose membrane (12–14 kDa molecular weight cutoff, Sigma Aldrich D9527) for 2 weeks at 40°C under magnetic stirring. Water was substituted twice a day to completely remove unreacted MAA.

In the case of pre-filtration, dialyzed GelMA solution was firstly filtered with laboratory filter paper or with a sequential filtration using laboratory filter paper and then 0.22 µm PES membrane filters (Aisimo, ASF33PS22S). Finally, GelMA was freeze-dried and stored at room temperature (RT) in the dark until use.

Zhu and colleagues previously used the second method (referred to as “Method 2”) to optimize the amount of MAA used and guarantee more consistent batch-to-batch results (B. H. Lee et al., 2015; Zhu et al., 2019). Briefly, 10 g of gelatin was dissolved at 10% w/v in a carbonate-bicarbonate (CB) buffer at 0.25 M and the pH was then adjusted to 9.4 with 5 M HCl (Sigma Aldrich, 320331) or NaOH (Sigma Aldrich, 221465) solutions. To reach the desired percentage of gelatin modification, different amounts of MAA were used: 0.938 mL, 0.705 mL and 0.317 mL for target DoF of 100% (i.e., High), 85% (i.e., Medium) and 60% (i.e., Low). Specifically, MAA was added slowly and dropwise, then the reaction proceeded for 1 h at 55°C under magnetic stirring at 500 rpm. After, the final pH was adjusted to 7.4 with small amounts of 5M HCl or NaOH solutions to stop the reaction. The solution was finally dialyzed against ddH_2_O with cellulose membrane (12–14 kDa molecular weight cutoff, Sigma Aldrich, D9527) for 1 week at 40°C under magnetic stirring. Water was substituted twice a day to completely remove unreacted MAA. GelMA was then freeze-dried and stored at RT in the dark until use.

### 2.2 Degree of functionalization (DoF)

The *o*-phtalaldheyde (OPA) based assay is considered a conventional technique to quantify the DoFs of photo-crosslinkable biopolymers (Pien et al., 2022) and has been used to characterize gelatin modification (Yue et al., 2017; Krishnamoorthy et al., 2019).

OPA reagent (Thermo Fischer, 26025) was warmed at RT before usage. Briefly, different solutions of unmodified gelatin in DPBS (0.02, 0.1, 0.5, 0.75 and 1 mg/mL) were prepared to derive the standard curve. GelMA solutions in DPBS were prepared at 1 mg/mL concentration. After proper dissolution by warming them at 50°C followed by vortexing, they were cooled down to RT and then reacted with OPA on a ratio of (1:2 v/v) for 60 s. A microplate reader (Synergy™ HTX Fluorescence Multi-Mode Microplate Reader) used an excitation/emission of 360/460 nm to measure the fluorescent intensity of the samples after 5 min from the reaction. The DoF was, then, calculated as described in the follow equation 1:

$$DoF=\left(1-\frac{C_{eq}}{C_{sample}}\;\right)∙100\%$$

Where $C_{eq}$ is the equivalent modified gelatin concentration of the sample determined by the standard curve and $C_{sample}$ is the tested sample concentration.

### 2.3 GelMAs and Pluronic formulation preparation

GelMA hydrogel was obtained by dissolving freeze-dried GelMA at a concentration of 10% w/v in Gibco BenchStable™ DMEM GlutaMAX^TM^ medium (Thermo Fisher, A41921-01), which had been previously combined with lithium phenyl-2,4,6-trimethylbenzoylphosphinate (LAP, Sigma Aldrich, 900889) as photoinitiator at the concentration of 2.5 mg/mL. This photoinitiator can be exited both by UV and blue light (Lim et al., 2020). The solution was heated at 60°C for 30 min and filtered sequentially through 0.45 µm and 0.22 µm PES membrane filters to sterilize it. All solutions were pre-warmed to 37°C before cells embedding.

Pluronic hydrogel was prepared by dissolving Pluronic F-127 (Sigma Aldrich, P2443) in ddH_2_O at a concentration of 32% w/v. To ensure proper mixing the solution was stirred at 300 rpm in a beaker cooled in an ice bath. The obtained sacrificial ink was filtered through 0.45 µm PES membrane filters (Aisimo, ASF33PS45S) and 0.22 µm PES membrane filters to sterilize it.

### 2.4 Swelling

To quantify the swelling behavior in different GelMA formulations, 400 μL of each 10% w/v GelMA solutions was casted in cylindrical molds with a 10 mm inner diameter and photopolymerized for 3 min in a UV oven (Asiga) at λ = 365 nm, 10 mW/cm^2^. Samples were then incubated at 37°C in DPBS for 24 h to reach swelling equilibrium. Afterward, excess water was gently removed from the hydrogels using paper and then weighed. Subsequently, the samples were freeze-dried and weighed again. The swelling ratio was calculated as described in equation 2:

$$Swelling\;ratio\;\left(\%\right)=\left(\frac{w_{s}}{w_{d}}-1\right)\%$$

Where $w_{s}$ is the swollen weight and $w_{d}$ is the freeze-dried weight.

### 2.5 Enzymatic degradation

The rate of GelMA degradation was evaluated through an enzymatic degradation assay. Briefly, 400 μL of each 10% w/v GelMA solutions with LAP was casted in cylindrical molds with a 10 mm inner diameter and photopolymerized for 3 min in a UV oven (Asiga) at λ = 365 nm, 10 mW/cm^2^.

Samples were freeze-dried to obtain the undegraded GelMA weight. Then, samples were rehydrated at 37°C in DPBS for 24 h to reach swelling equilibrium. A collagenase solution in DPBS at 0.75 U/ml was prepared using a type XI collagenase (Sigma Aldrich, C9407). Finally, each swollen sample was immersed in 1.5 ml of collagenase solution, collected at every time point (0h, 4h, 6h, 8h, 24h), and freeze-dried to measure the degraded GelMA weight. The residual weight was measured as:

$$Residual\;Weight\;\left(\%\right)=\left(\frac{w_{2}}{w_{1}}\right)\%$$

Where $w_{2}$ is the degraded weight and $w_{1}$ is the undegraded weight.

### 2.6 Rheological characterization

Rheological measurements were performed using an Anton Paar rheometer (Physica MCR 302) in parallel-plate mode with a 0.3 mm gap between two aluminum plates, each with a diameter of 20 mm. To determine the linear viscoelastic range (LVE), oscillatory tests were performed at a constant frequency of 1 Hz, ranging from a strain of 0.01%–1000%. A temperature ramp test was performed to measure the storage modulus (G′) of GelMAs and Pluronic F-127 at a constant frequency of 10 Hz and a constant strain 1% ranging from 3°C to 37°C with a linear increase of 2°C per minute. Based on the results obtained, a constant temperature of 12°C was used for GelMAs derived from type B gelatin and 20°C for GelMAs derived from type A gelatin for further tests, regardless of their DoFs.

Shear thinning behavior was tested by setting the shear rate range from 1 to 1,000 s^−1^. Assessment of the viscosity recovery, after shear stress application, was obtained by applying a shear rate of 1 s^−1^ for 25 s of 100 s^−1^ for 50 s, and again 1 s^−1^ until the end of the measurement.

Real-time photorheological measurements were performed using a dedicated quartz bottom plate. An optic fiber LED light l = 405 nm and 10 mW/cm^2^ was placed under the bottom plate. A constant strain of 5% and a constant angular frequency of 10 rad/s were set, and the UV light was switched on after 30 s to let the system stabilize before the photopolymerization.

All experiments were carried out in the LVE and at least three times.

### 2.7 Cell culture

For cell culture analysis, two human lung adenocarcinoma cell lines, A549 and A549-GFP^+^, kindly provided by Dr. Valentina Monica, Department of Oncology, University of Torino, AOU San Luigi Gonzaga were used. For A549-GFP^+^, A549 were infected to constitutively express histonic protein H2B fused with the Green Fluorescent Protein (GFP). Both cell lines were cultured in Gibco BenchStable™ RPMI 1640 GlutaMAX^TM^ medium (Thermo Fisher, A41923-01) supplemented with 10% v/v Fetal Bovine Serum (Sigma Aldrich, F9665) and 1% v/v penicillin/streptomycin (Sigma Aldrich, P4333) and incubated in a humidified incubator at 37°C with 5% CO_2_.

### 2.8 Cell viability Assessment

Viability of A549 cells was determined using a 3-(4,5-Dimethylthiazol-2- il)-2,5-Diphenyltetrazolium Bromide (MTT, Sigma Aldrich, M2128) assay (Bhattacharyya et al., 2022). Briefly, 1.5 × 10^6^ cells/mL were embedded in GelMA dissolved in DMEM at a concentration of 10% w/v and 50 μL volume was cast in a 96-well tissue culture plate (Greiner Bio-one, 651160). After, GelMA was crosslinked for 1 min using a UV oven (Asiga) at λ = 365 nm, 10 mW/cm^2^ and incubated with 200 μL of complete medium. At the desired time points, after medium removal, samples were incubated at 37°C for 4 h with 200 μL of MTT solution at the concentration of 0.5 mg/mL in cell culture medium. Then, the reaction was stopped by the removal of the solution and formazan salts were dissolved in 200 μL of DMSO (Sigma Aldrich) by shaking the plate at 80 rpm for 2 h at RT. Absorbance was measured at 570 nm, using 650 nm as reference wavelength, by BioTeck Synergy™ HTX Multi-Mode Microplate Reader.

To assess post-printing viability, bioprinted constructs were stained with Live/Dead kit (Sigma Aldrich, 04511). Briefly, the solution for the reaction was prepared according to the manufacturer’s instruction. After two washing steps in DPBS of 5 min each, the staining solution was added and incubated at 37°C for 30 min. The constructs, after being washed with DPBS, were analyzed by a microscope (Eclipse Ti2 Nikon, Tokyo, Japan) equipped with a Crest X-Light spinning disk confocal microscope and a Lumencor SPECTRA X light engine.

### 2.9 3D bioprinting

The different bioprinted architectures were designed using the BioCAD software (RegenHu, Switzerland) and produced with a 3D discovery bioprinter (RegenHu, Switzerland).

GelMA containing cells (1.5 × 10^6^ cells/mL) was loaded in 3 mL UV-secure cartridges (Sigma Aldrich, 928801-1EA) and cooled in ice for 10 min to allow an initial pre-gelation of the hydrogels. In the meanwhile, Pluronics F-127 sacrificial ink was loaded in 5 mL clear cartridges (Sigma Aldrich, 928836-1EA) and allowed to warm up at RT.

The formulation was extruded using temperature-controlled pneumatically driven extrusion printhead (RegenHu) equipped with 25G conical nozzles with an internal diameter of 250 μm (Nordson EFD, 916765), directly into 12 well suspension plates (Greiner Bio-one, 665102). The feed rate was 20 mm/s with a printing pressure of 0.080–0.100 MPa for GelMA derived from Type A gelatin and 0.050–0.080 MPa for GelMA derived from Type B Gelatin. The cartridge temperature was set at 20°C and 12°C, whereas the cooling plate temperature was set at 18°C and 4°C for GelMA derived from type A gelatin and for GelMA derived from type B gelatin, respectively.

Pluronic F-127 was extruded at RT using a pneumatically driven extrusion printhead (RegenHu) equipped with a 27G blunt needle with an internal diameter of 200 μm (Nordson EFD, 917532). The feed rate was 10 mm/s with a printing pressure of 0.4-0.45 MPa.

Each printed structure was photopolymerized for 2 min using an optic fiber LED light λ = 405 nm and 10 mW/cm^2^ and covered with complete medium.

### 2.10 Cell seeding of the channels

After printing and photopolymerization, the plate was cooled down at 4°C for 15 min. Afterwards, the sacrificial ink was washed away with DPBS and then, A549 GFP^+^ cells were resuspended in complete medium at the density of 1.5 × 10^6^ cells/mL. Finally, 10 µL of the prepared mix were added inside the empty channel, the well was filled with 1.5 mL of complete medium and incubated at 37°C, 5% CO_2_ until the analysis performed by microscopy.

### 2.11 Statistical analysis

Statistical analysis was performed by using Two-Way ANOVA and *t*-test, depending on the need. The corresponding symbols were used in the graphs shown to indicate the *p*-value, none = *p* > 0.05, * = *p* ≤ 0.05, ** = *p* ≤ 0.01, *** = *p* ≤ 0.001, **** = *p* ≤ 0.0001.

## 3 Results and discussion

### 3.1 Pre-filtration effects on GelMA chemical, mechanical and biological properties

**FIGURE 1:**
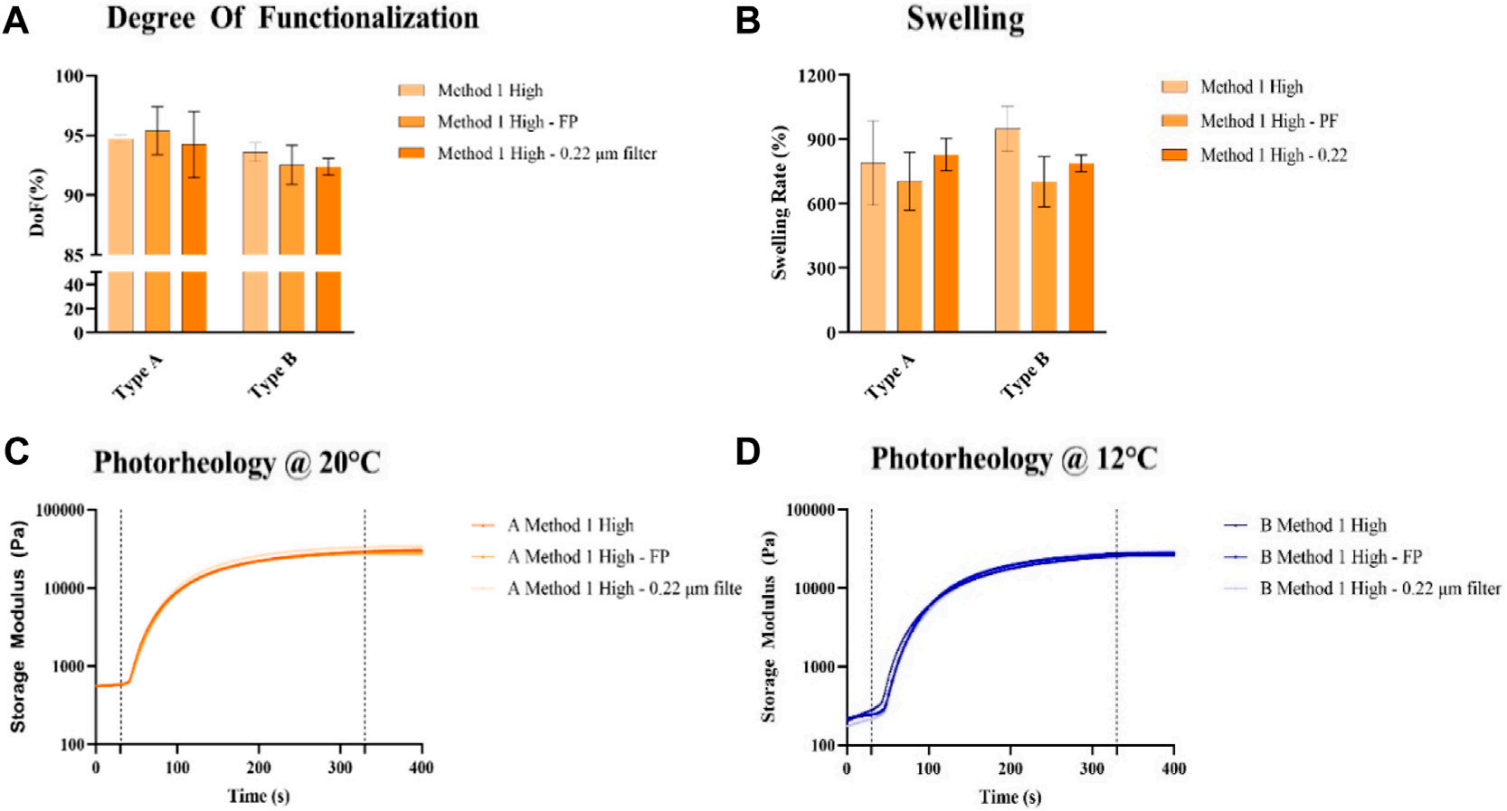
**(A)** DoF quantification of GelMA obtained using Method 1, with or without pre-filtration, **(B)** Swelling rate of GelMA obtained using Method 1 derived from type A and type B gelatin at 10%, with or without pre-filtration after 1 day after reaching the swelling aequilibrium **(C, D)**, Photoreological characterization of GelMA obtained using Method 1 derived from type A **(C)** and type B **(D)** gelatin at 10%, with or without pre-filtration at the optimized temperatures for each gelatin source.

Filtration of GelMA solution prior freeze-drying is commonly used in literature, both for Method 1 synthesis (Ko et al., 2019; Zhou et al., 2022) and for Method 2 synthesis protocols (Shirahama et al., 2016; Zhu et al., 2019)**.** As the method of filtration is not fully described in literature, two commonly used methods were tested, first with laboratory paper filter (PF), then with a combination of laboratory paper filter and 0.22 μm membrane filters. To verify the potential beneficial effects of this preliminary step, a chemical, mechanical, and biological characterization, initially with GelMA obtained using Method 1 and with a High target DoF, was conducted, No statistical difference was found in the DoFs (Figure 1A), and GelMA High showed a DoF >90% in all cases obtained with the Method 1 synthesis. Swelling is one of the pivotal properties of hydrogels, as it indicates their ability to increase in volume by absorbing solvents. In the case of GelMA, the swelling equilibrium is reached after 24 h (He et al., 2023), whereas the swelling rate is mainly influenced by DoF and concentration. According to our experiments, filtration of the dialyzed GelMA solutions does not statistically affect the swelling rate (Figure 1B). Photorheological tests (Figures 1C, D) are used to evaluate the mechanical properties of the crosslinked hydrogels and were conducted at different temperatures for each GelMA source, considered to be optimal for the printing process of each derived bioink (20°C for GelMA derived from type A gelatin, 12°C for GelMA derived from type B gelatin) based on a temperature ramp test (Supplementary Figure S1). A similar behavior was observed among GelMA obtained from the same source, reaching a G′ of 27.7 ± 1.3 kPa for GelMA derived from Type B gelatin and 31.6 ± 2.0 kPa for GelMA from Type A gelatin, confirming that filtration of the dialyzed solutions does not affect the mechanical properties of the photopolymerized hydrogels.

**FIGURE 2:**
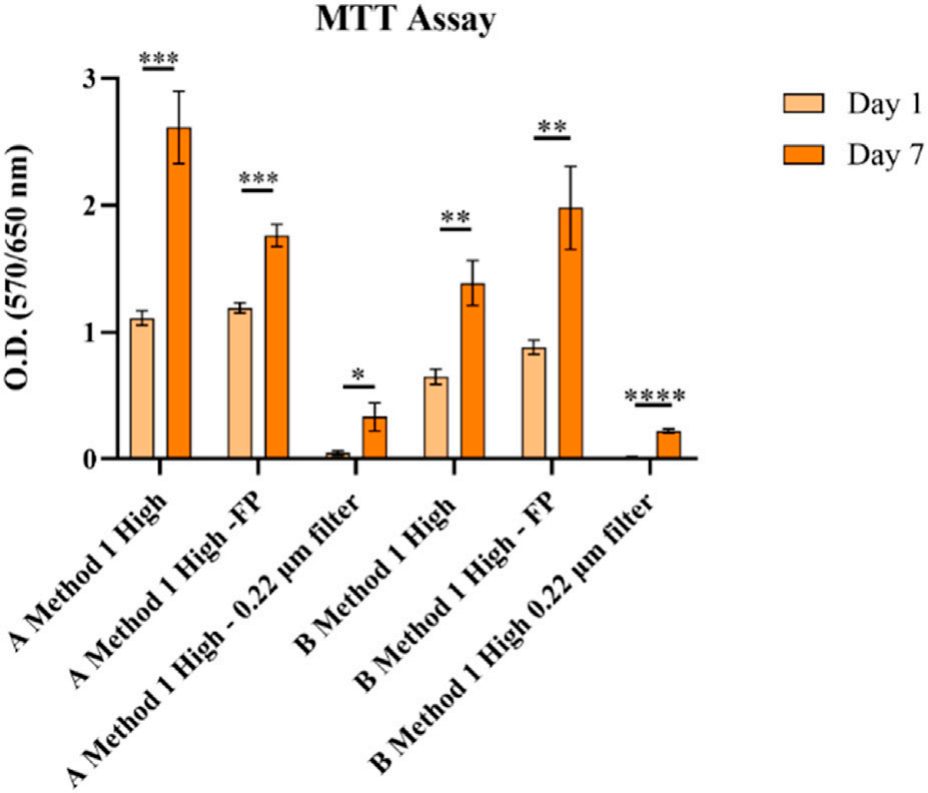
MTT analysis in A549 cells on GelMA obtained using Method 1. None = *p* > 0.05, * = *p* ≤ 0.05, ** = *p* ≤ 0.01, *** = *p* ≤ 0.001, **** = *p* ≤ 0.0001.

The MTT assay on A549 cells (Figure 2) showed that GelMA without pre-filtration and with laboratory filter paper pre-filtration supports cell viability, whereas pre-filtration with 0.22 μm membrane filters greatly hindered it. Based on the results obtained from this characterization, pre-filtration of GelMA before freeze-drying was not further used for GelMA syntheses regardless of the method.

### 3.2 Evaluation of gelatin source and methacrylic anhydride amount effects of GelMA hydrogel properties

**FIGURE 3:**
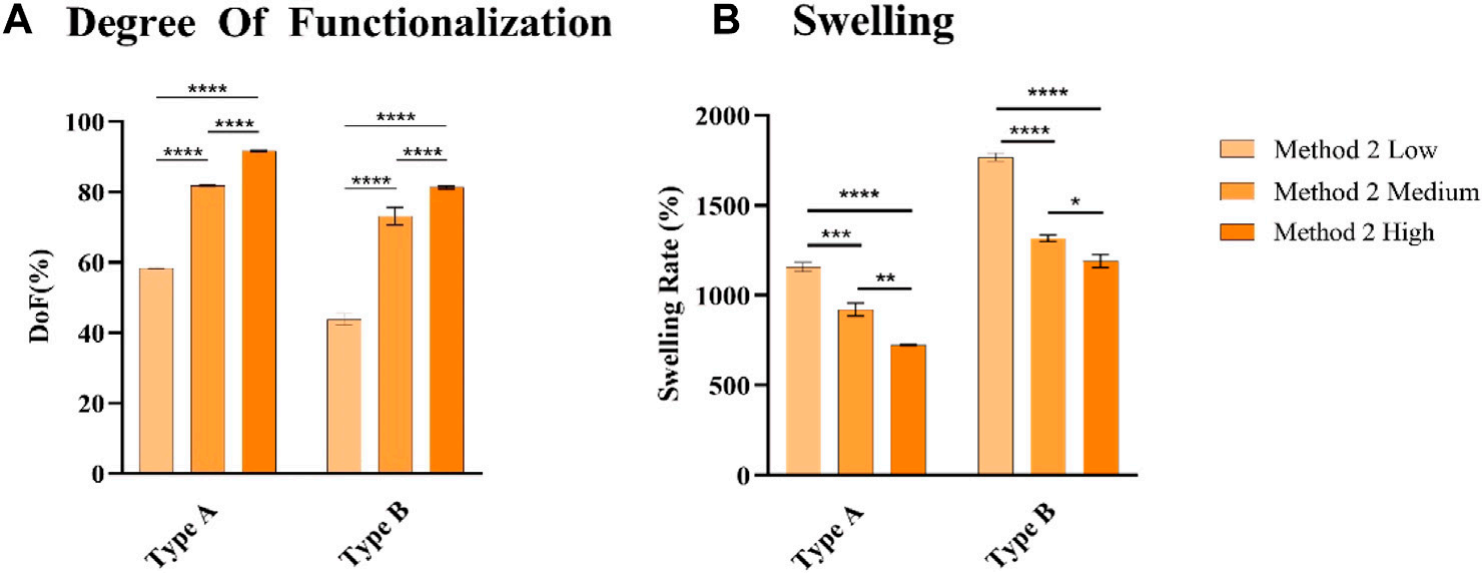
**(A)** DoF quantification of GelMA derived from type A and type B gelatin **(B)**, Swelling rate of GelMA obtained derived from type A and type B gelatin at 10%, after 1 day after reaching the swelling equilibrium. None = *p* > 0.05, * = *p* ≤ 0.05, ** = *p* ≤ 0.01, *** = *p* ≤ 0.001, **** = *p* ≤ 0.0001.

From the chemical characterization, GelMA obtained from Type A gelatin showed a higher DoF than GelMA from Type B gelatin (Figure 3). Low, Medium, and High DoF resulted in 58.37% ± 0.29%, 81.84% ± 0.17%, 91.66% ± 0.17% and 43.80% ± 1.59%, 73.14% ± 2.49%, 81.31% ± 0.42% for Type A and B derived GelMA, respectively. For examples, GelMA derived from Type A gelatin with a medium target DoF and GelMA derived from Type B gelatin with a high target DoF possess similar values 81.84% ± 0.17% and 81.31% ± 0.42%, respectively. As previously reported in literature, those difference has to be attributed to the different isoelectric points (IEP) of Type A and B gelatin, respectively pH 7-9 for Type A gelatin and 5-6 for Type B gelatin (Aljaber et al., 2023). The One-Pot method proposed in literature (Zhu et al., 2019), optimizes the quantity of MAA used adjusting the pH based on Type A gelatin IEP. This finding underlines the necessity to optimize the MAA quantity and reaction pH to reach a desired target DoF taking into account the gelatin source especially when using Method 2 protocols which involves a more efficient control of the pH during the synthesis reaction. As higher DoFs results in tighter polymeric networks due to the increased number of crosslinks (Krishnamoorthy et al., 2019), swelling rates of the hydrogels (Figure 3B) decreased as the DoF increases, ranging from 724% ± 3% to 1158% ± 25% for GelMA derived from Type A gelatin and from 1190% ± 36% to 1768% ± 22% for GelMA derived from Type B gelatin.

**FIGURE 4:**
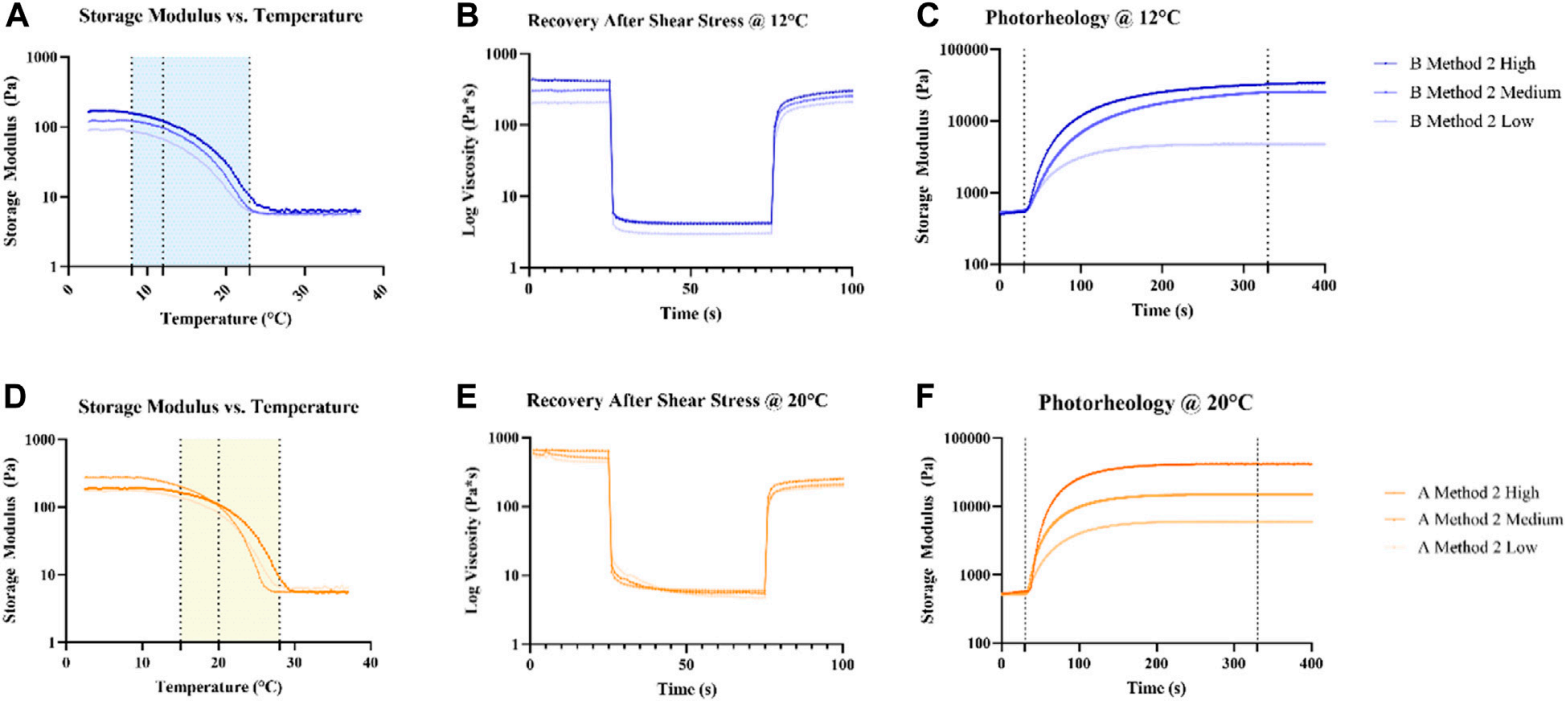
Rheological **(A, B, D, E)** and photorheological **(C, F)** characterization of GelMA derived from Type A and B gelatin using Method 2 synthesis. Temperature ramp tests were used to identify a suitable printing temperature for each source of GelMA, marked with a dotted line. The following tests were performed at the choesen temperature for each source.

An extensive rheological characterization of different GelMAs formulations has been carried on. One crucial aspect to assess is the optimal printing temperature for each GelMA source. A temperature ramp test (Figures 4A, D) was performed on GelMA derived from Type A and B gelatin with different DoFs synthetized with Method 2. GelMA derived from Type A showed a temperature transition range from gel to sol from 15°C to 28°C, whereas GelMA Type B exhibited the same interval between 8°C and 25°C. The optimal compromise to have good printability and a sufficiently high temperature that does not significantly hinder cell viability was found to be 12°C for GelMA derived from Type B gelatin and 20°C for GelMA from Type A gelatin. In this way, a G’ > 100 Pa was obtained from all GelMA formulations, except for GelMA from Type B gelatin with a Low DoF, that, however, was found inadequate to handle even after photopolymerization. Then, amplitude sweep and flow curve measurements (Supplementary Figure S2) provided the LVE range to perform the following characterization and confirming the thixotropic behavior of GelMA hydrogels. A sufficient recovery rate after high shear stress is crucial for a good bioink candidate (Paxton et al., 2017) as the deposed bioink filament must retain its shape without collapsing. As reported (Figures 4B, E) GelMA derived from Type A gelatin exhibits a low recovery rate, reaching ∼40% of the initial viscosity after 20 s regardless its DoF, whereas GelMA derived from Type B gelatin demonstrated a much better recovery, reaching 73%, 90% and 98% of the initial viscosity for High, Medium, and Low DoF respectively. An explanation of this behavior can be attributed to the fact that Type B gelatin is completely denatured, resulting in generally shorter gelatin chains, while Type A gelatin generally has residual collagen triple helix structures (Gorgieva and Kokol, 2011) that may contribute to a slower recovery rate. On the other hand, photorheological tests (Figures 4C, F) underlined the dependency between the DoF and the photopolymerized hydrogels G′. This is advantageous as, after the choice of the gelatin source, hydrogels with different stiffness can be obtained by optimizing just a set of printing parameters.

**FIGURE 5:**
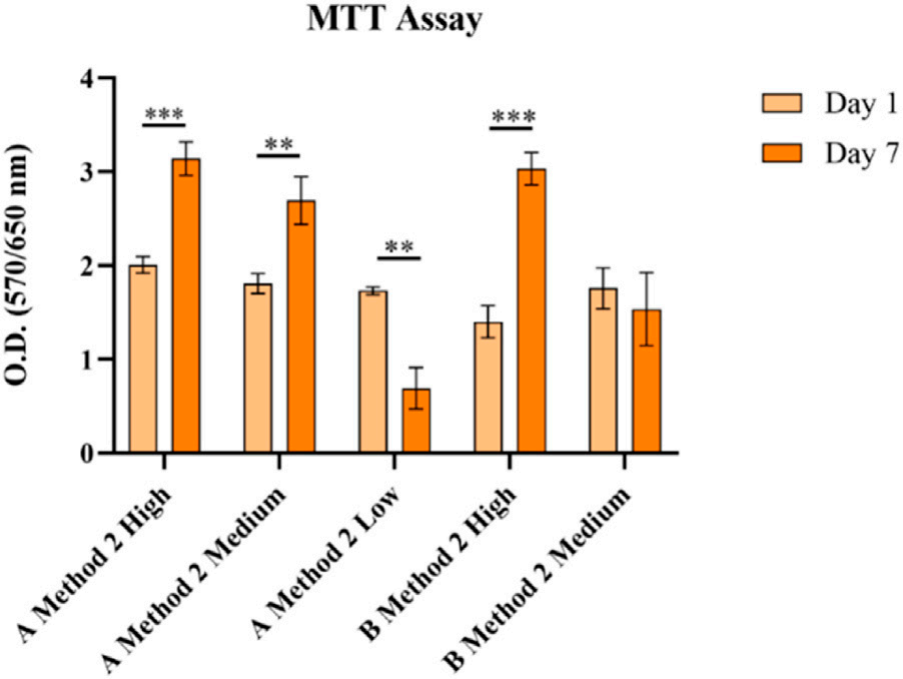
MTT analysis in A549 on different GelMA formulations with different DoFs and gelatin sources obtained using Method 2 synthesis. None = *p* > 0.05, * = *p* ≤ 0.05, ** = *p* ≤ 0.01, *** = *p* ≤ 0.001, **** = *p* ≤ 0.0001.

Results from the MTT assay performed on A549 cells (Figure 5) showed good ability to sustain cell viability. During the experiment, excessive swelling, and degradation of GelMA derived from Type B gelatin with a Low DoF made it impossible to handle the samples, thus results are not shown. Also, cells embedded in GelMA derived from Type A with a Low DoF tended to escape from the hydrogel and adhere/proliferate on the well bottom in a 2D fashion.

### 3.3 Synthesis protocols comparison

**FIGURE 6:**
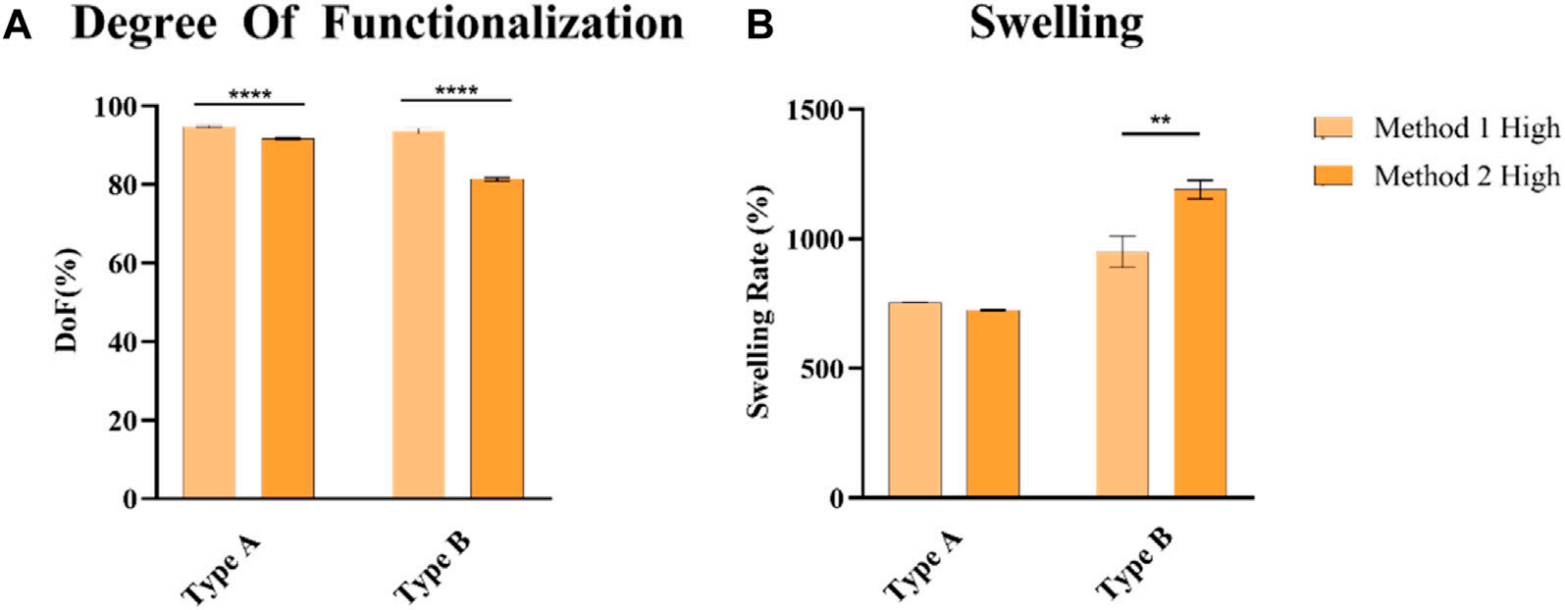
**(A)** DoF quantification of GelMA derived from type A and type B gelatin **(B)**, Swelling rate of GelMA obtained derived from type A and type B gelatin at 10%, after 1 day after reaching the swelling equilibrium. None = *p* > 0.05, * = *p* ≤ 0.05, ** = *p* ≤ 0.01, *** = *p* ≤ 0.001, **** = *p* ≤ 0.0001.

A comparison between the two synthesis protocols was performed on GelMA derived from Type A and B gelatin with a High DoF, as it is found to be the most widely used in literature for bioprinting applications due to its ability to maintain good printability and promotion of cell proliferation (W. Liu et al., 2017; Ning et al., 2020; Schulik et al., 2023; Villata et al., 2023). As indicated by DoF measurements (Figure 6A), GelMA derived from Type A gelatin exhibits DoFs in the range between 90% and 95%, 94.73% ± 0.31% and 91.66% ± 0.17% for Method 1 and 2 syntheses, respectively, which can be considered similar in terms of performance. The results of this analysis were confirmed by a not-statistically significant difference in the swelling rate measurements (Figure 6B). Different results were obtained with GelMA derived from Type B gelatin, reaching 93.60% ± 0.77% and 81.31% ± 0.42% for Method 1 and 2 syntheses, respectively, indicating a >10% difference in the DoF. Also, the swelling rate of Method 1 and 2 GelMA formulations derived from Type B gelatin showed a statistically relevant difference, 950% ± 61% and 1190% ± 36% for Method 1 and 2, respectively, in line with the results obtained with DoF measurements. As previously discussed, the different results obtained when using Type B gelatin may be attributed to its different IEP (Aljaber et al., 2023), thus not being suitable to obtain the same results as Method 1 synthesis when using parameters found in literature relative to Method 2 synthesis protocol that uses Type A gelatin as source for GelMA.

**FIGURE 7:**
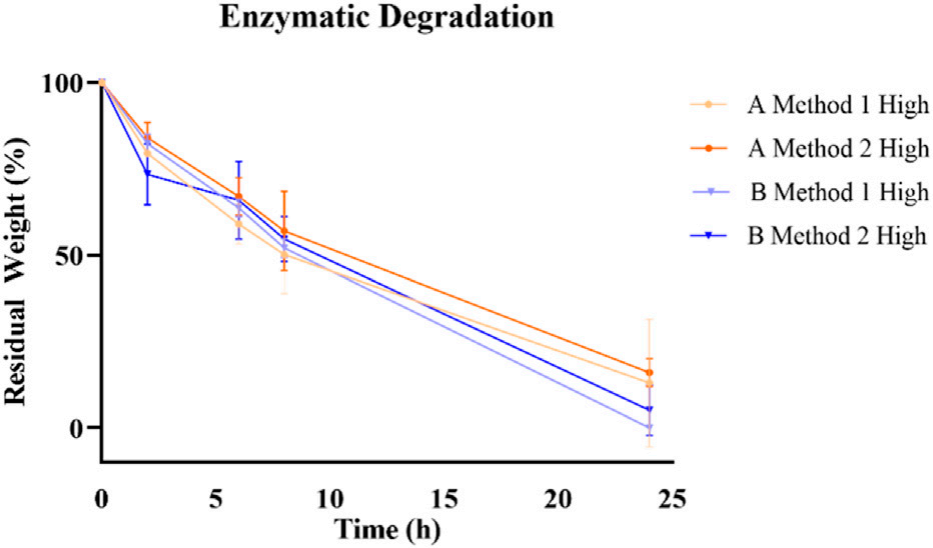
Enzymatic degradation profile of different GelMA formulation over 24 h.

Enzymatic degradation profiles (Figure 7) exhibited a similar fashion for all the formulations with only Method 1 GelMA derived from type B gelatin fully degraded after 24 h.

**FIGURE 8:**
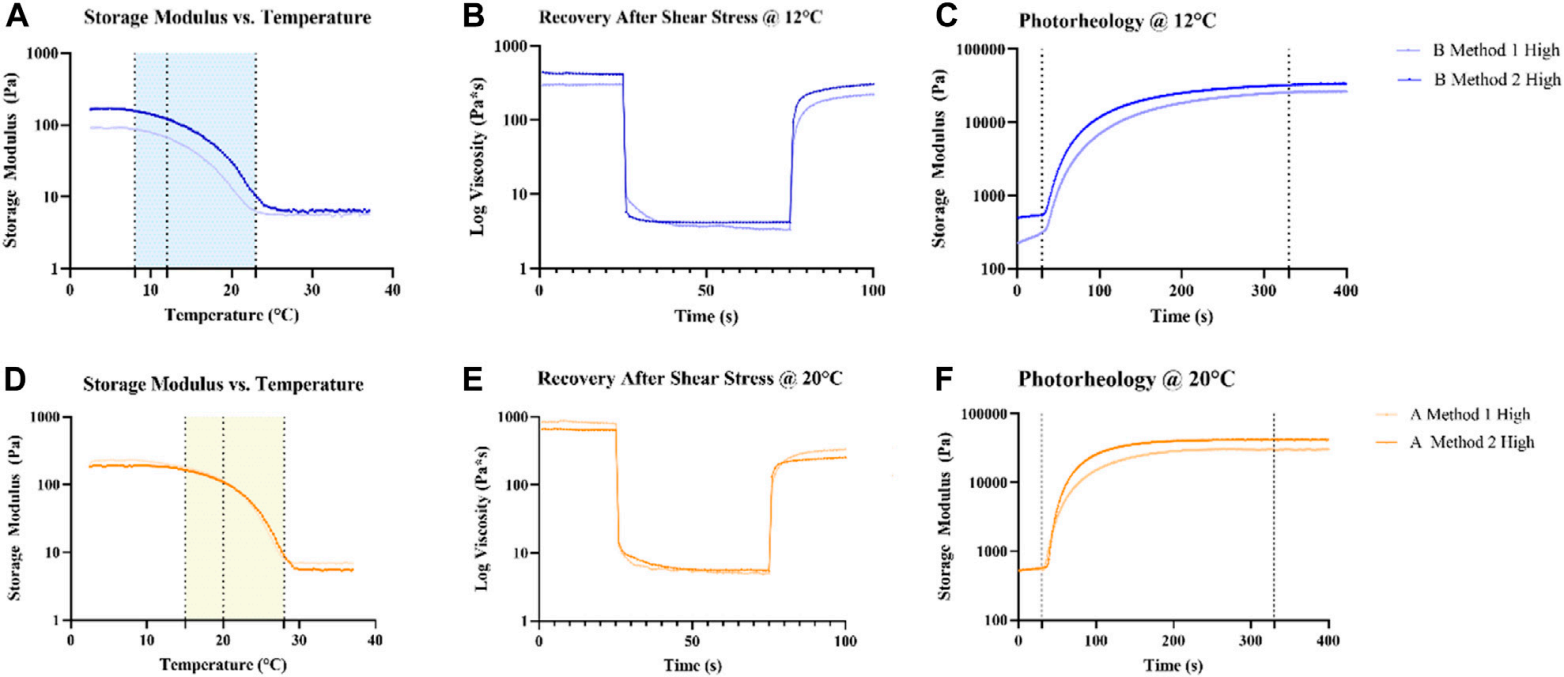
Comparison of the rheological **(A,B,D,E)** and photorheological **(C,F)** properties of GelMA derived from Type A and B gelatin using Method 1 and 2 syntheses. Temperature ramp tests were used to identify a suitable printing temperature for each source of GelMA, identified with a dotted line. The following tests were performed at the chosen temperature for each source.

Comparison of the rheological characteristics of GelMA obtained with different protocols has been carried out similarly as reported in the previous paragraph. Temperature ramp tests (Figures 8A, D A, D) showed no difference in the behavior of GelMA derived from Type A gelatin, whereas GelMA derived from Type B gelatin revealed a marked difference in the viscosity exhibited during the gel state, with Method 1 GelMA having less than half G′ than Method 2 GelMA (70.45 ± 0.97 Pa vs. 165,73 ± 5.6 Pa). This difference may cause lower printing resolution in Method 1 GelMA derived from Type B gelatin. Recovery capacity (Figures 8B, E), on the other hand, maintained the same trend in both syntheses, with 40% and 42% recovery after 20 s for GelMA derived from Type A gelatin with Method 1 and 2, respectively, and 73% for GelMA derived from Type B gelatin with both Method 1 and 2. Also, photorheology tests (Figures 8C, F) demonstrated comparable trends and values in both syntheses.

**FIGURE 9:**
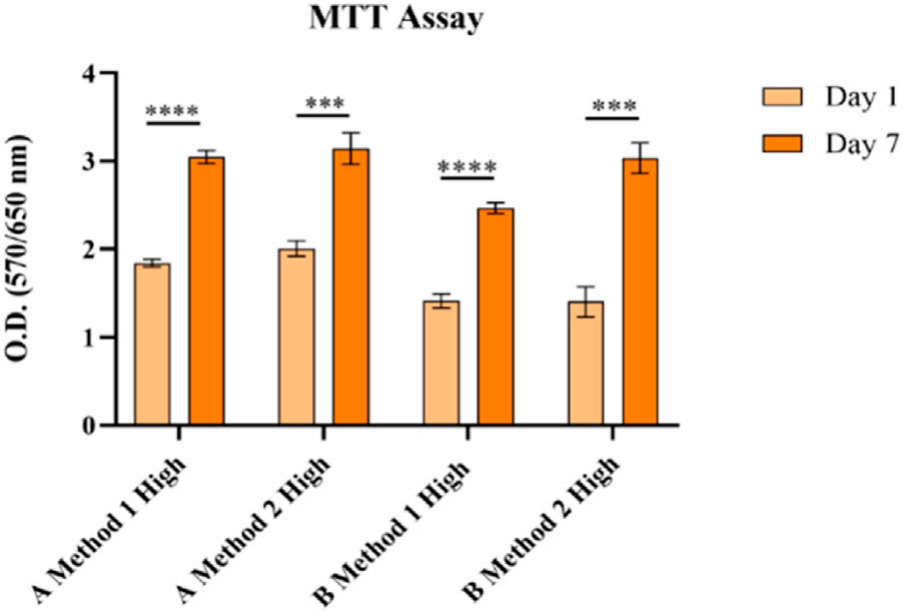
MTT analysis in A549 cells on different GelMA formulations with different gelatin sources and a High DoF obtained using Method 1 and 2 syntheses. None = *p* > 0.05, * = *p* ≤ 0.05, ** = *p* ≤ 0.01, *** = *p* ≤ 0.001, **** = *p* ≤ 0.0001.

The MTT assay on A549 cells (Figure 9) showed that all the formulations guaranteed an increase in cell viability suggesting cell proliferation has taken place.

### 3.4 GelMA 3D bioprinting

**FIGURE 10:**
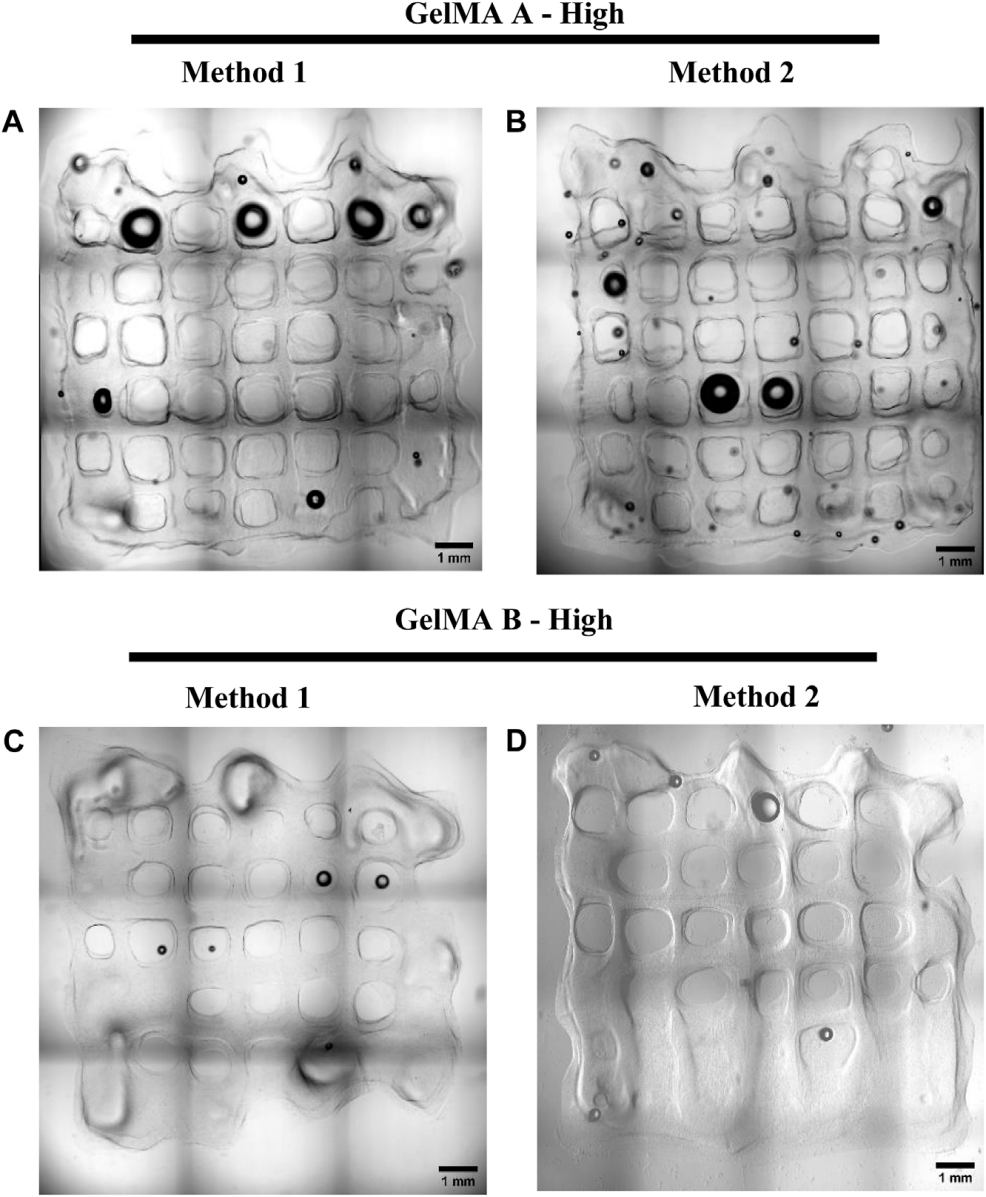
Confocal microscopy brightfield images of A549-embedded GelMA grids, GelMA derived from Type A gelatin with High DoF obtained with Method 1 and 2 (**(A, B)**, respectiviely) and GelMA derived from Type B gelatin with a High DoF obtained with Method 1 and 2 (**(C, D)**, respectively). Scalebar = 1 mm.

For the bioprinting printing experiments, only High DoF GelMA formulations were used, as high DoF is generally used in different tissue engineering applications (Krishnamoorthy et al., 2019). Grids were printed with 10 layers with 1.5 mm interspace between the struts, for a dimension of 10 × 10 × 2.5 mm. Average strut width was measured from confocal microscopy images (Figure 10). GelMA derived from Type A gelatin (Figures 10A, B) displays thinner struts, with a width of 288 ± 73 µm for Method 1 synthesis and 288 ± 56 µm for Method 2 synthesis, whereas GelMA derived from Type B gelatin (Figures 10C, D) displayed, when intact, thicker struts, with a width of 435 ± 89 µm for Method 1 synthesis and 403 ± 111 µm for Method 2 synthesis.

**FIGURE 11:**
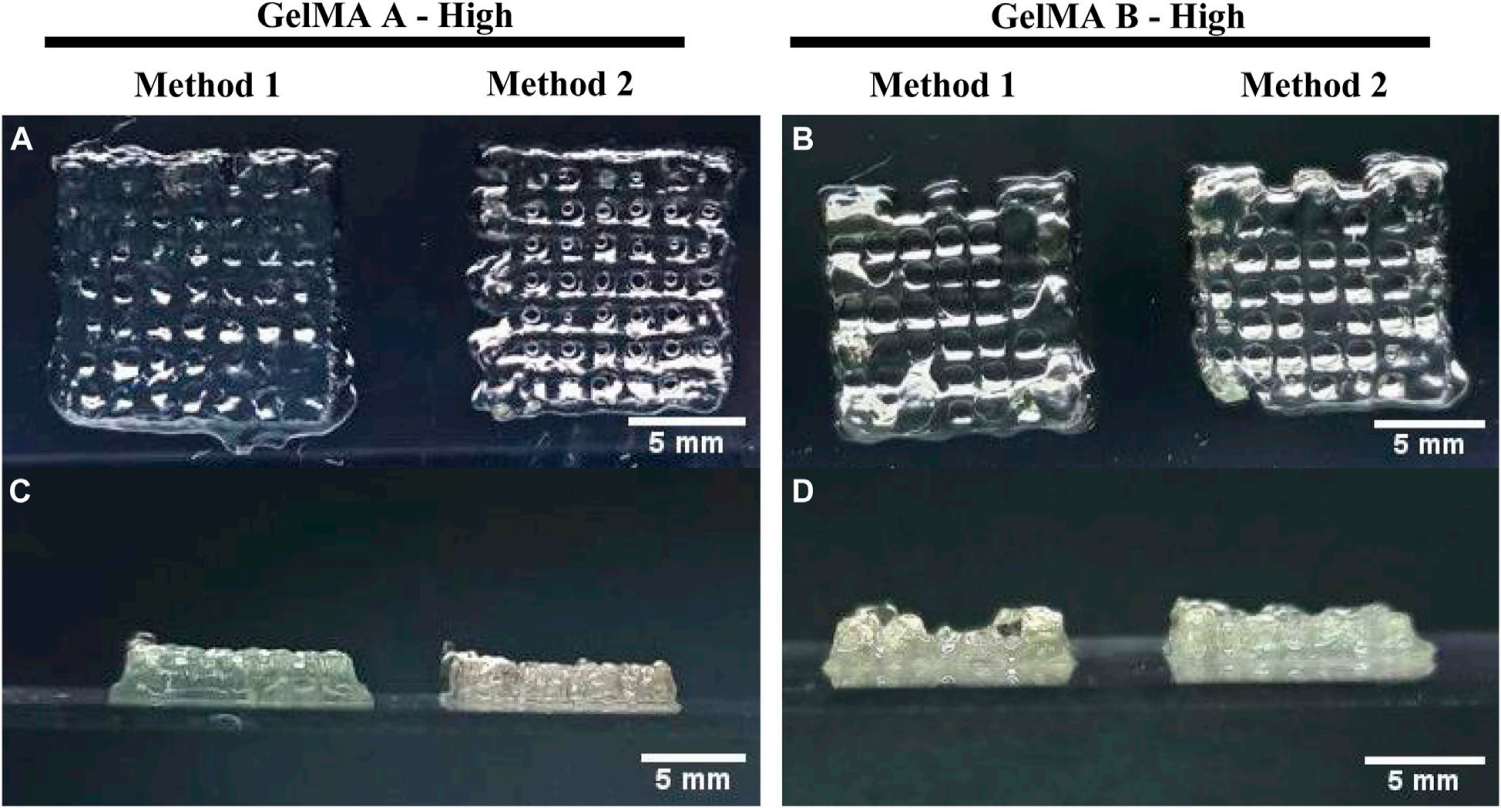
3D Bioprinting of A549-embedded GelMA grids. Lateral and front view of GelMA derived from Type A gelatin with High DoF obtained using Method 1 and 2 **(A, C)** and GelMA derived from Type B gelatin with High DoF obtained using Method 1 and 2 **(B, D)**. Scalebar = 5 mm.

Moreover, GelMA type A has shown the ability to support thicker structures. GelMA derived from Type A gelatin obtained with Method 2 maintained the desired shape on the bottom layers, avoiding the elephant foot effect that has been observed with the other formulations (Figures 11C, D).

**FIGURE 12:**
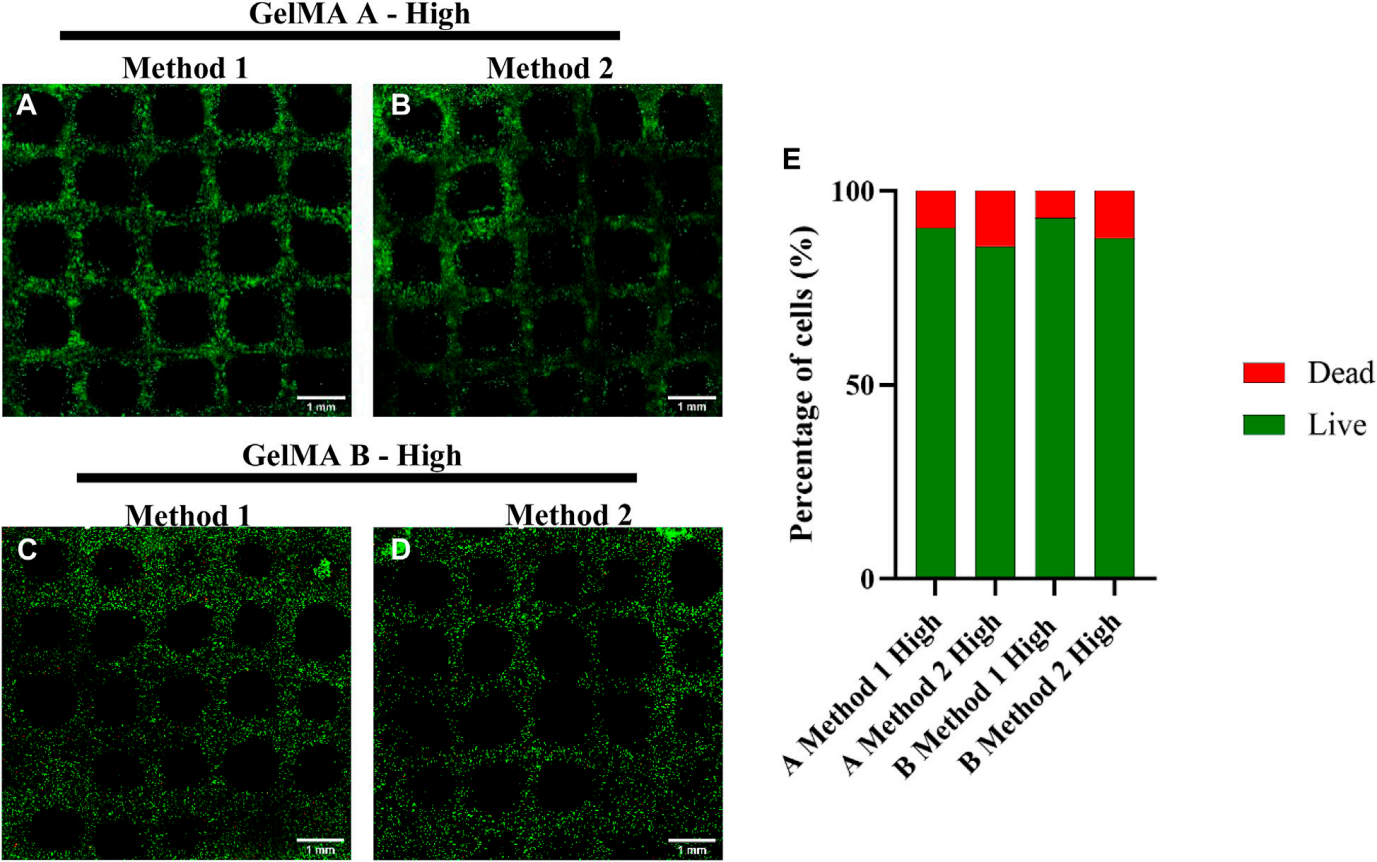
Live & Dead Staining of A549-embedded GelMA grids, GelMA derived from Type A gelatin with High DoF obtained with Method 1 and 2 (**(A, B)**, respectively) and GelMA derived from Type B gelatin with a High DoF obtained with Method 1 and 2 (**(C, D)**, respectively). Live & Dead of different formulations quantification **(E)**. Scalebar = 1 mm.

Live & Dead staining was performed right after the printing process to assess print-related cell death and all the formulation showed a viability >85%. As shown in Figure 12, GelMA obtained with Method 1 allowed to maintain higher viability regardless of the gelatin source.

Based on those results, GelMA derived from Type A gelatin obtained with Method 2 was considered the formulation with the best compromise between high printing fidelity and high cell viability.

### 3.5 GelMA 3D multimaterial printing

**FIGURE 13:**
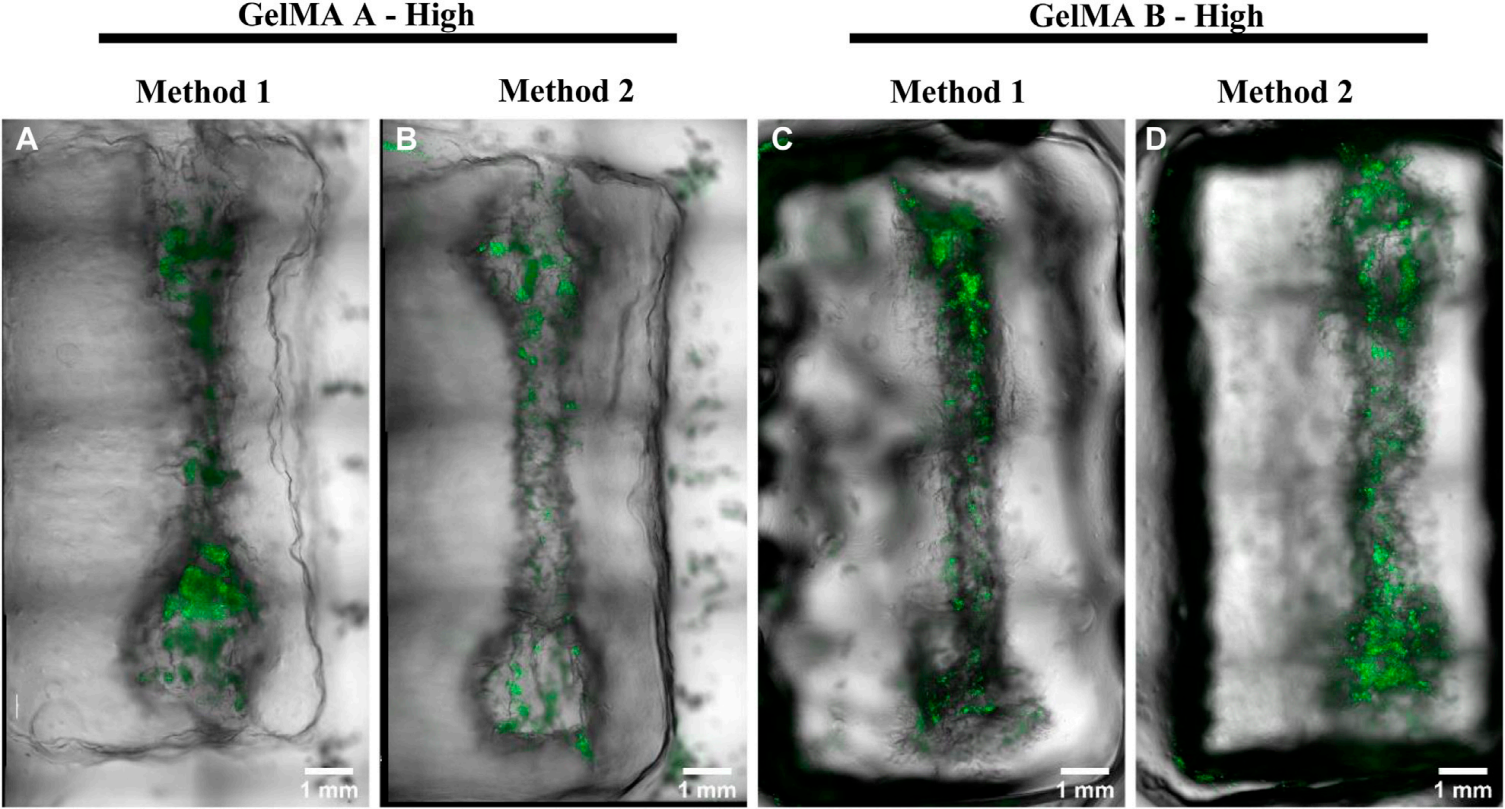
Seeded channels in printed GelMA derived from Type A gelatin with High DoF obtained with Method 1 and 2 (**(A, B)**, respectively) and GelMA derived from Type B gelatin with a High DoF obtained with Method 1 and 2 (**(C, D)**, respecti respectively). A549 GFP^+^ cells were seeded in the hollow channels 1 day before acquiring the images. Scalebar = 1 mm.

For multimaterial printing, 13 × 6 × 20 mm rectangular construct with 0.5 mm interspace between the struts were printed. A single channel embedded in the GelMA structure was printed with Pluronic F-127. Pluronics F-127 was removed and channels were seeded with A549 GFP^+^ as previously reported. After 1 day of culturing images were taken (Figure 13). Overall, all channels demonstrated a sufficient perfusability, however, only GelMA from gelatin Type A synthesized with Method 2 showed a better shape fidelity and allowed reproducibility over sequential constructs printing in a single session (Supplementary Figure S3).

## 4 Conclusion

This study compared the effects of the synthesis protocol and gelatin source on GelMA bioink formulations, with an orientation towards multimaterial bioprinting. Method 2 synthesis showed overall better performances than Method 1 with Type A gelatin as source, uses optimized amounts of MAA and has shorter dialysis time that allows faster syntheses. For multimaterial printing applications, GelMA derived from Type A gelatin obtained with Method 2 synthesis was found to be the best biomaterial candidate for our bioink, showing appropriate viability support and good interactions with the sacrificial material Pluronics F127 to obtain soft models containing hollow, patent channels that can be further optimized to form a microvasculature. Performances from GelMA derived from Type B gelatin varied significantly when comparing the synthesis protocols, further studies to optimize its target DoF are mandatory to optimize its synthesis with the Method 2 protocol, as its consistency respect to Method 1 protocol is not respected as with GelMA derived from Type A gelatin. However, since GelMA derived from Type B exhibited great ability to maintain cell viability, its use as bioink base would be strongly recommended for thin constructs or, if a thicker model is needed, embedded bioprinting, a 3D bioprinting strategy that exploits sacrificial baths to maintain the bioink shape during the extrusion process, could represent a solution.

## Data Availability

The original contributions presented in the study are included in the article/Supplementary Material, further inquiries can be directed to the corresponding author.
