# Supplementary material for: GelMA synthesis and sources comparison for 3D multimaterial bioprinting
Source: Front Bioeng Biotechnol. 2024 Mar 25;12:1383010. doi: 10.3389/fbioe.2024.1383010 (PMC10999536; doi:10.3389/fbioe.2024.1383010)
Supplement: Supplementary file 1 [file DataSheet1.docx]

**Supporting information**

**GelMA synthesis and sources comparison for 3D multimaterial bioprinting**

Cesare Gabriele Gaglio^1^, Désireé Baruffaldi^1^, Candido Fabrizio Pirri^1,2^, Lucia Napione^1^, Francesca Frascella^1*^

*Corresponding author: [francesca.frascella@polito.it](mailto:francesca.frascella@polito.it)

^1^ DISAT - PolitoBIOMed Lab – Politecnico di Torino, Corso Duca degli Abruzzi 24, 10129 Turin, Italy

^2^ Center for Sustainable Future Technologies, Italian Institute of Technology, Via Livorno 60, 10144 Turin, Italy

**Fig.S1** Temperature ramp test comparing GelMA derived from Type A or B gelatin obtained with Method 2 protocol with High DoF and Pluronics F127 thermosensitive behavior.


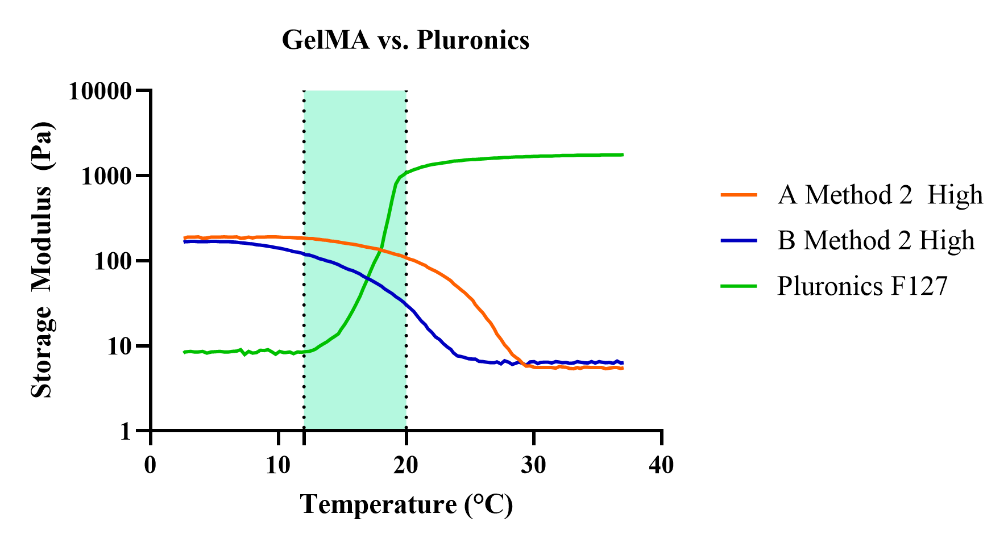


**Fig.S2** Preliminary rheological characterization of different GelMA formulations to determine the LVE region and assess thixotropic behavior.


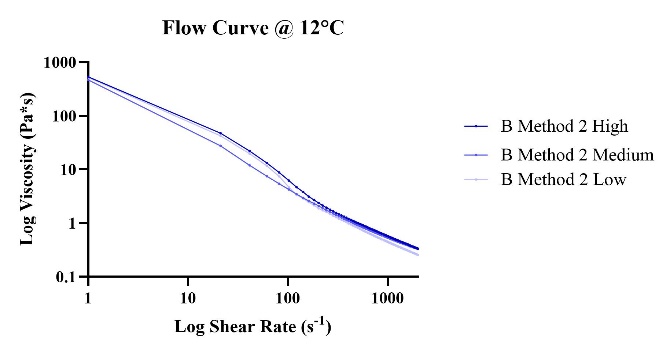

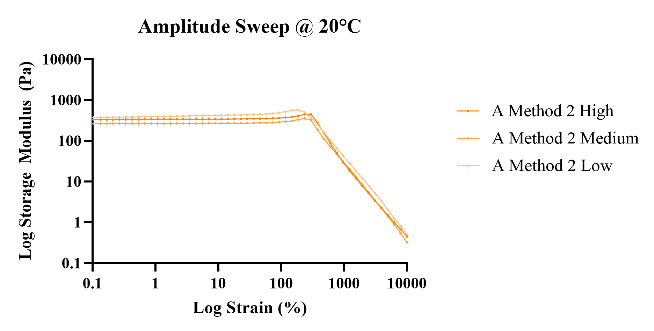

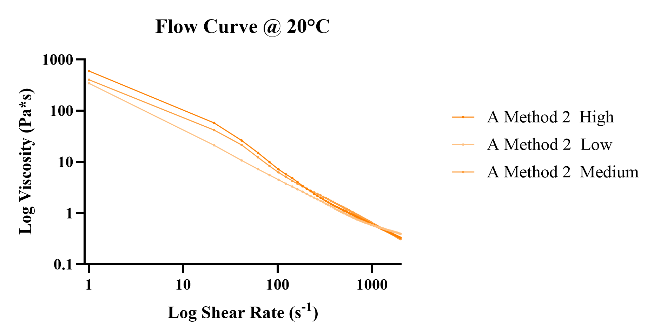

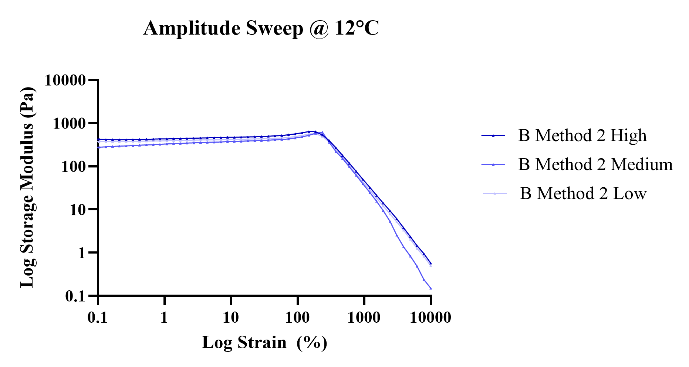

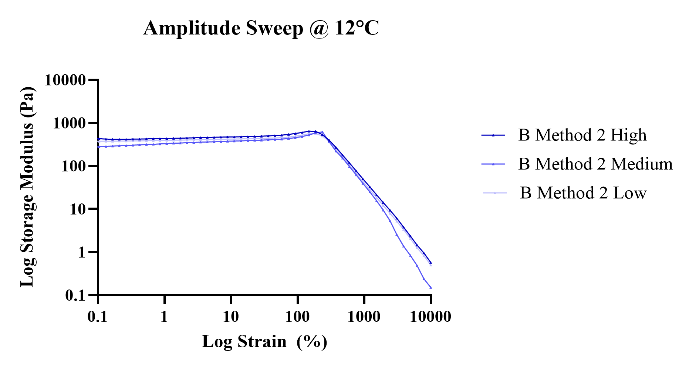

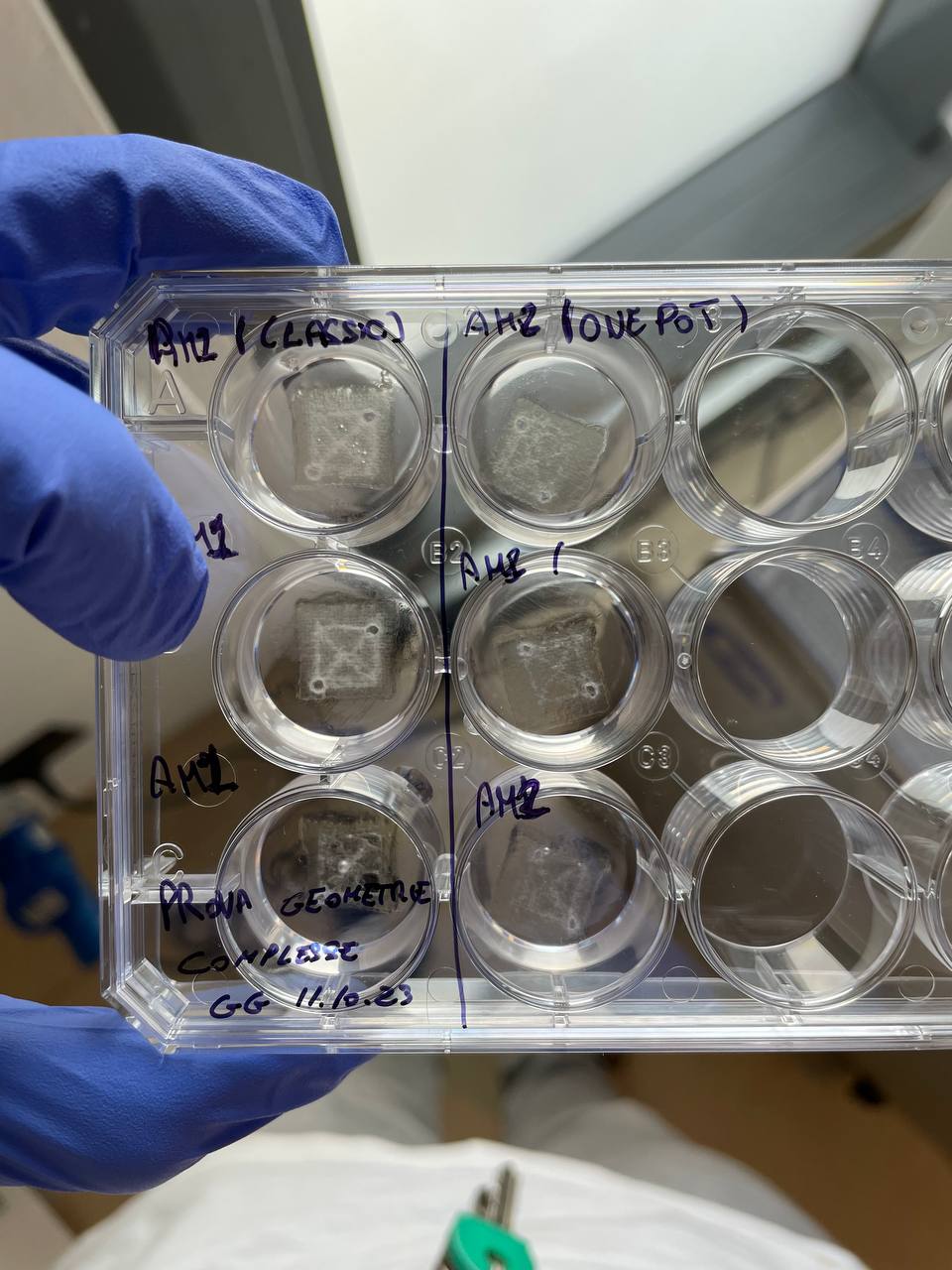

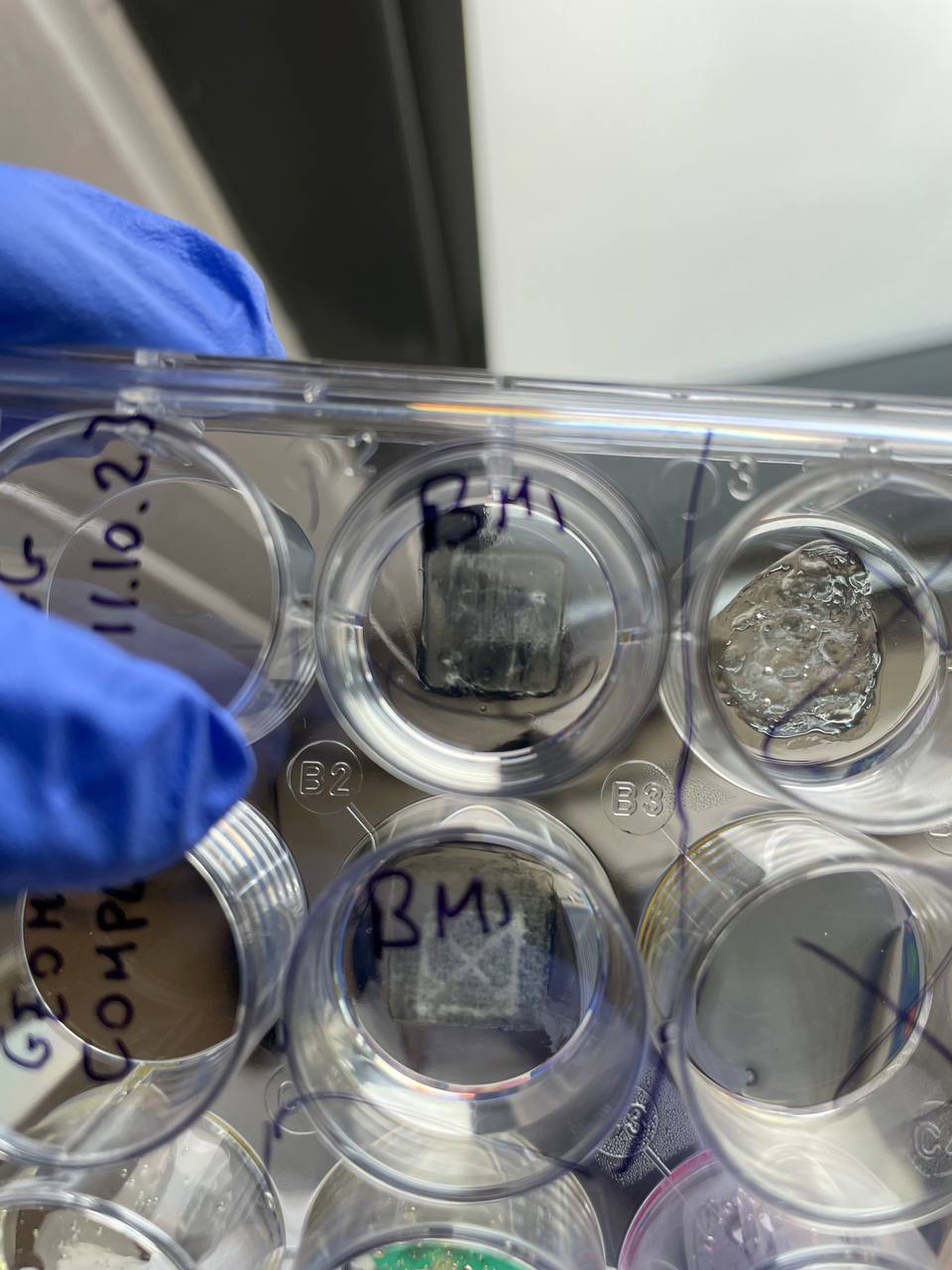

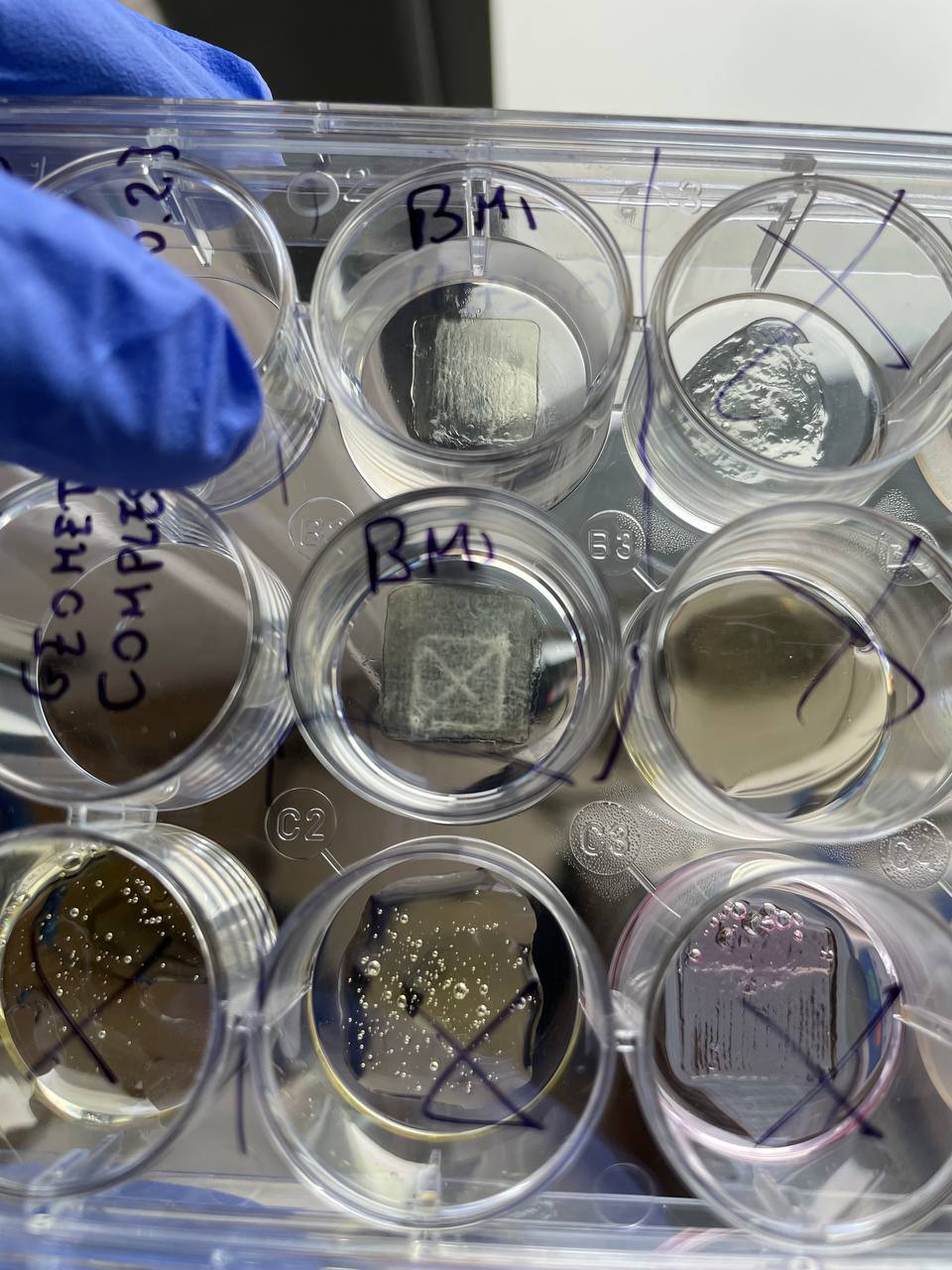

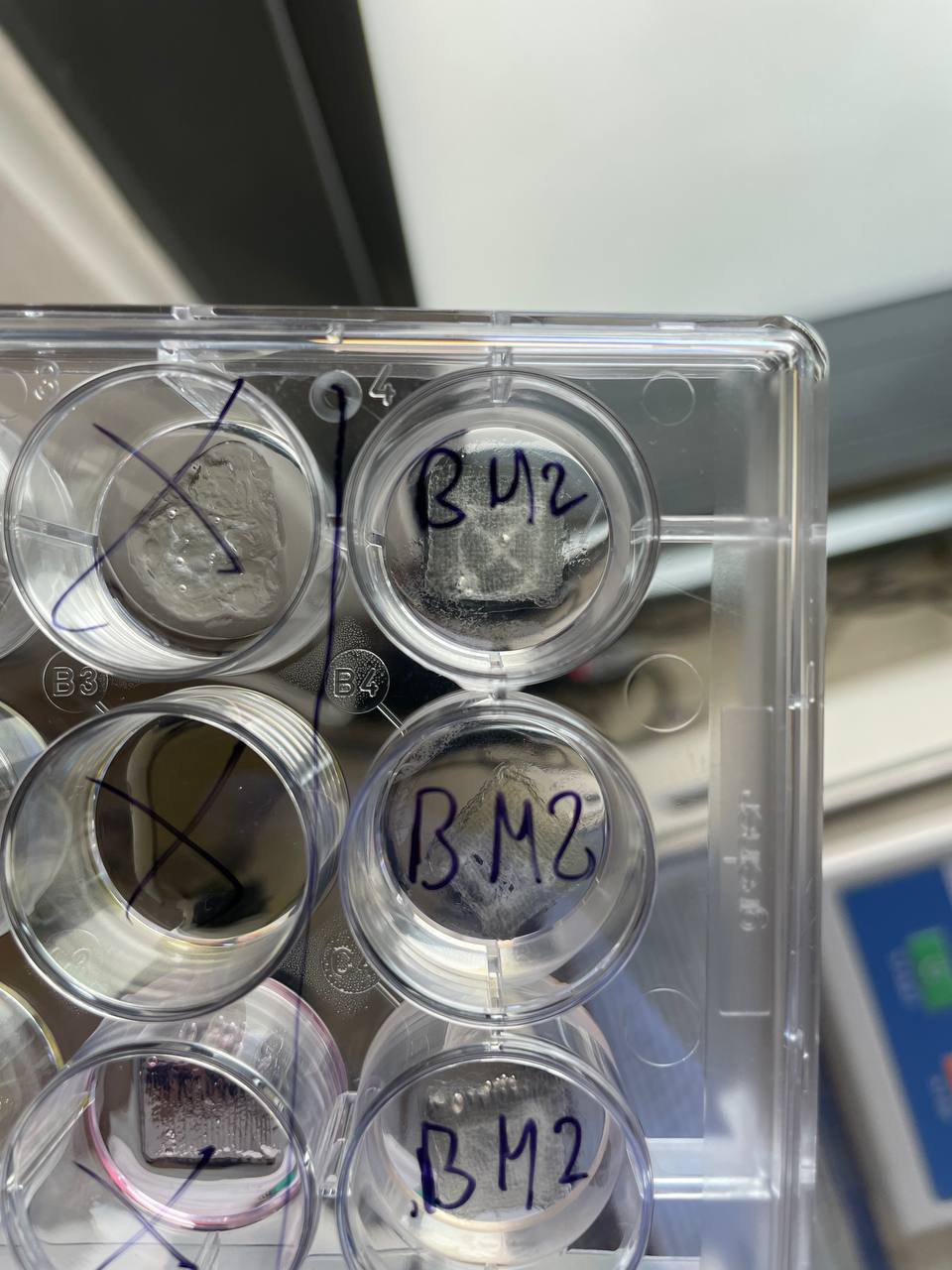

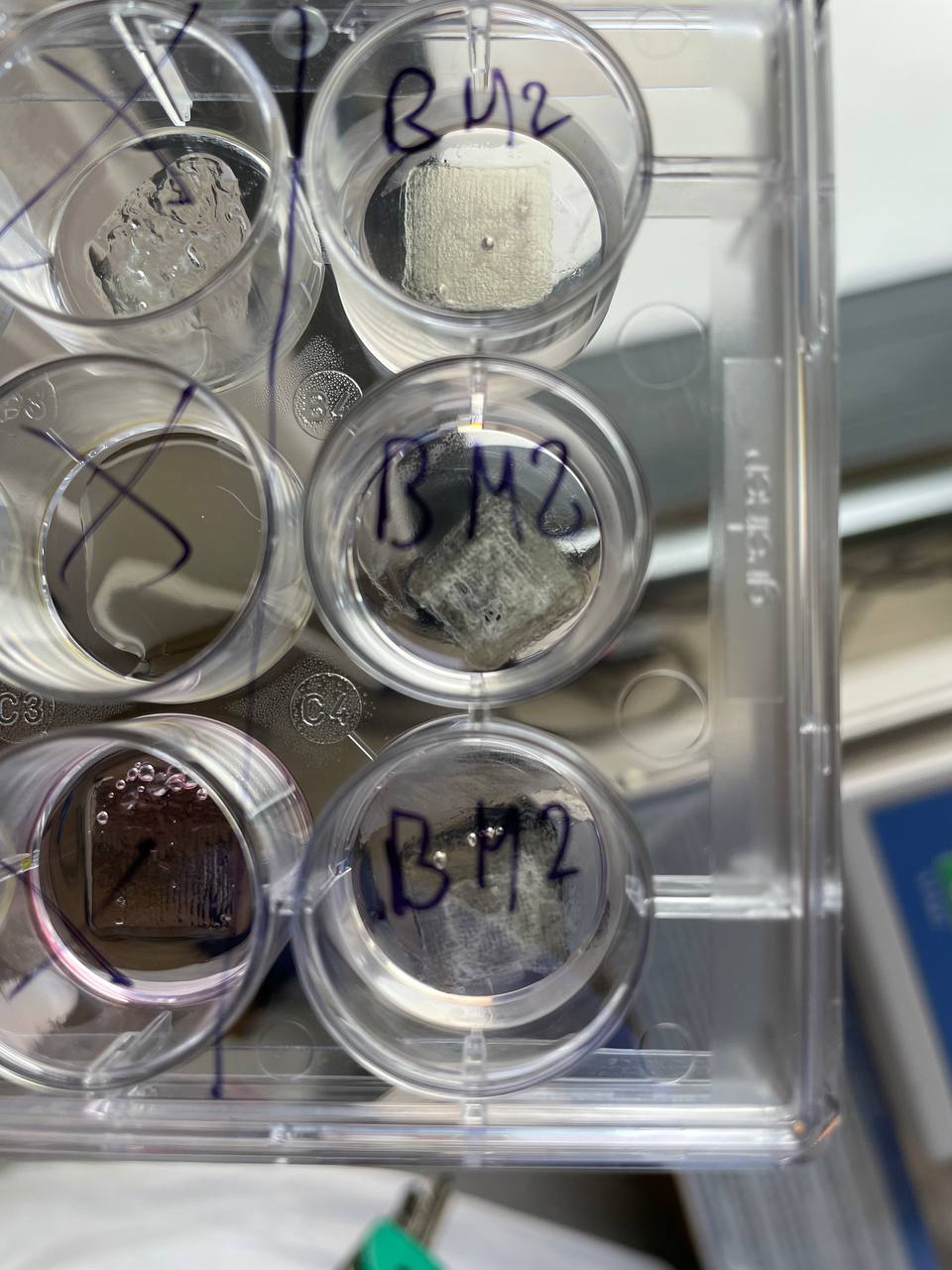

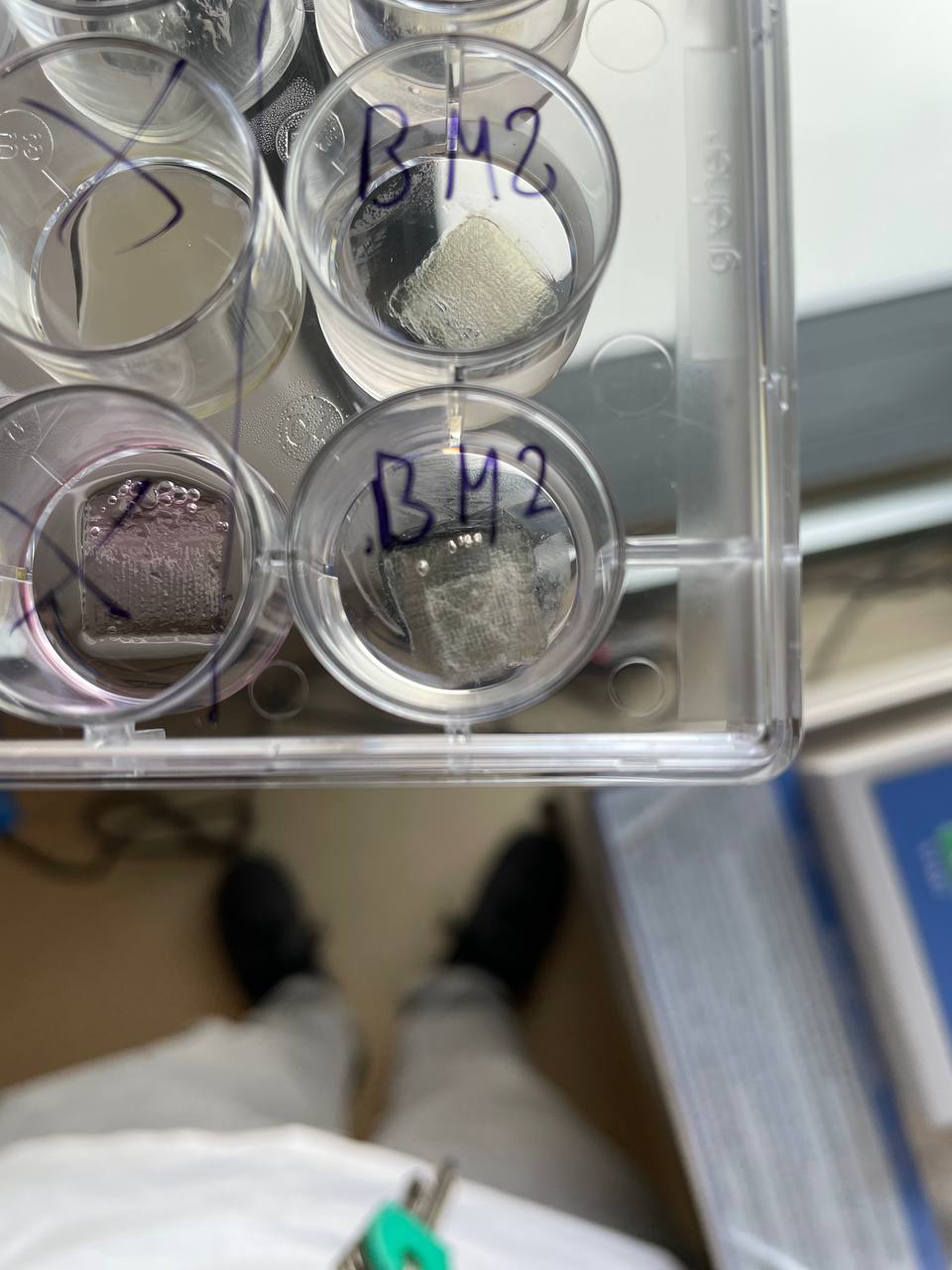


Sequential printing

**Fig. S3** Complex channel printing with different GelMA formulations and Pluronic F127 generated channels
